# Foot Sole Contact Forces vs. Ground Contact Forces to Obtain Foot Joint Moments for In-Shoe Gait—A Preliminary Study

**DOI:** 10.3390/s23156744

**Published:** 2023-07-28

**Authors:** Joaquín L. Sancho-Bru, Enrique Sanchis-Sales, Pablo J. Rodríguez-Cervantes, Carles Vergés-Salas

**Affiliations:** 1 Department of Mechanical Engineering and Construction, Universitat Jaume I, 12071 Castellón de la Plana, Spain; cervante@uji.es; 2 Departmental Section of Podiatry, Nursing Department, Universitat de València, 46010 Valencia, Spain; enrique.sanchis-sales@uv.es; 3 Departmental Section of Podiatry, Department of Clinical Sciences, Faculty of Medicine and Health Sciences, Universitat de Barcelona, 08907 L’Hospitalet de Llobregat, Spain; cverges@ub.edu

**Keywords:** force plate, pressure platform, instrumented insoles, multisegment kinetic foot model, in-shoe model

## Abstract

In-shoe models are required to extend the clinical application of current multisegment kinetic models of the bare foot to study the effect of foot orthoses. Work to date has only addressed marker placement for reliable kinematic analyses. The purpose of this study is to address the difficulties of recording contact forces with available sensors. Ten participants walked 5 times wearing two different types of footwear by stepping on a pressure platform (ground contact forces) while wearing in-shoe pressure sensors (foot sole contact forces). Pressure data were segmented by considering contact cells’ anteroposterior location, and were used to compute 3D moments at foot joints. The mean values and 95% confidence intervals were plotted for each device per shoe condition. The peak values and times of forces and moments were computed per participant and trial under each condition, and were compared using mixed-effect tests. Test–retest reliability was analyzed by means of intraclass correlation coefficients. The curve profiles from both devices were similar, with higher joint moments for the instrumented insoles at the metatarsophalangeal joint (~26%), which were lower at the ankle (~8%) and midtarsal (~15%) joints, although the differences were nonsignificant. Not considering frictional forces resulted in ~20% lower peaks at the ankle moments compared to previous studies, which employed force plates. The device affected both shoe conditions in the same way, which suggests the interchangeability of measuring joint moments with one or the other device. This hypothesis was reinforced by the intraclass correlation coefficients, which were higher for the peak values, although only moderate-to-good. In short, both considered alternatives have drawbacks. Only the instrumented in-soles provided direct information about foot contact forces, but it was incomplete (evidenced by the difference in ankle moments between devices). However, recording ground reaction forces offers the advantage of enabling the consideration of contact friction forces (using force plates in series, or combining a pressure platform and a force plate to estimate friction forces and torque), which are less invasive than instrumented insoles (which may affect subjects’ gait).

## 1. Introduction

The study of foot kinetics has been emphasized as an important tool for identifying, assessing and treating foot disorders [1,2]. First, the kinetics of the ankle joint affected by specific pathologies or after having been treated was studied by using models in which the foot was considered a unique segment, created from data recorded with motion capture systems and force plates (e.g., [3,4,5]). More recently, multisegment foot models have been proposed [6,7,8], whose main differences lie in the foot segmentation considered along with the number and location of markers and, therefore, the joints that can be addressed with each model [9]. One of the main difficulties in creating a multisegment foot model is the segmentation of ground reaction forces because force plates measure the total contacting force applied at the center of pressure (CoP) of the foot. To address this problem, Bruening et al. [6] proposed using two consecutive force plates placed side by side. To analyze a particular joint, the subject under study has to step in such a way that the distal segment comes into contact with the anterior platform, and the proximal segment does so with the posterior platform. However, this segmentation alternative has some drawbacks: it hinders subjects’ natural gait because of platform targeting; it is difficult to ensure the perfect alignment of the joint with the platforms; and it requires repeating the measurement for each joint under study. Another applied alternative is using pressure platforms instead of force plates [10,11,12]. In this case, segmentation is easier because it only requires identifying which platform cells are distal to each joint at every time point. The drawback of this alternative lies in only the normal reactive component being measured. Some works estimate friction components by superimposing the measurements from a pressure platform and a force plate [7,8,13,14,15]. However, they assume proportionality between the friction force and torque with the normal contact force in a given contacting area. Although this is not rigorous, errors for the planovalgus condition have been found to be less than 3% [8]. The ankle moments reported in the literature with and without considering frictional forces [16,17] are similar, and some studies suggest that not contemplating frictional forces does not significantly affect joint torques [11,18] because frictional forces are much smaller than the normal components of reactive forces during gait, and because of the differences in the moment arms of normal and frictional forces.

All these models were developed for the gait analysis under barefoot conditions, which makes the clinical application of the results difficult because humans use shoes to walk. Moreover, different orthotic treatments are commonly prescribed for different pathologies to modify moments at foot joints and to avoid pressure points [19,20,21,22,23]. Consequently, research into how orthoses interfere with ankle joint mechanics has grown in the last decade [24,25,26,27,28,29]. However, there are very few studies about their effect on midtarsal (MT) and metatarsophalangeal (MP) joints, and only some kinematic studies [30] because of the difficulties with performing in-shoe gait analysis. In-shoe gait analysis still requires a boost [13]. The first attempts to develop multisegment in-shoe foot models focused on the kinematic aspect by addressing the placement of markers. Two alternatives have been proposed to either fix markers directly on the shoe sole or upper [31] or fix them directly on the skin using holes or ‘windows’ made in the shoe upper [32]. Both alternatives involve some errors. If markers are fixed on the shoe, shoe marker sets do not represent foot movement because of the motion of the foot inside the shoe, and also because of shoe deformation. In the event of using holes, the changes made to the shoe may make the shoe behave unrealistically, and hole sizes need to be controlled to avoid damaging the shoe [33,34].

However, no work has addressed the in-shoe kinetic analysis of foot joints, except for the ankle [35]. Obviously, joint moments for the ankle can be computed directly from the ground forces measured with a force plate. These forces represent all the distal forces that act on the joint, which are equal to the resultant one of all the contact forces between the foot and shoe. However, this is not so straightforward for other foot joints, such as the MT of MP joints, because the way that the contact forces between the foot sole and the ground (the forces measured with either a force plate or a pressure platform) are finally distributed as contact forces between the foot and the shoe is complex, and also depends on specific shoe features. One alternative is directly using the contact forces between the foot and the foot sole by means of employing instrumented insoles. However, these instrumented insoles only provide information about the contact forces between the sole of the foot and the insole of the shoe, while the shoe is in contact all over the foot, generating contact forces that may also affect the foot joint moments; they may also affect gait because of the interposition of the insole film inside the shoe, which affects the friction coefficient (and, therefore, foot motion inside the shoe) and comfortability.

This paper presents a preliminary study that aims to analyze the impact of considering foot sole contact forces (measured with instrumented insoles) vs. ground contact forces (measured with a pressure platform) on foot joint moments (ankle, MT and MP joints) during in-shoe gait. The study provides novel quantitative data on the differences between the two alternatives, and delves into the sources of these differences. The aim is to identify the pros and cons of using the different common available sensors in gait laboratories and to establish the research needs of new sensors for developing more accurate and advanced multisegment in-shoe foot models.

## 2. Materials and Methods

### 2.1. Participants

The experiment was carried out with 10 participants (5 females and 5 males), aged 39 ± 11 years and weighing 71 ± 12 kg. Inclusion criteria included subjects between 18 and 60 years without a history of neuromuscular problems, diabetes or foot or ankle surgery. Subjects using orthotics or reporting pain in the lower extremity were excluded. All the participants provided written informed consent to participate in the study, which was approved by the University’s Ethical Committee (reference number CD/90/2021).

### 2.2. Experiment Description

**Figure 1:**
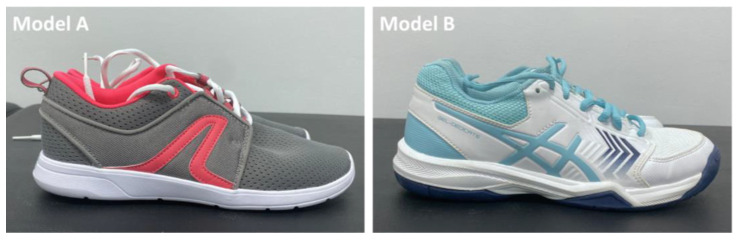
Shoes used for the experiment. Model A is a lightweight flexible shoe meant for walking; Model B is a pair of sneakers for playing tennis.

The participants were asked to walk at a self-selected speed along a 7 m walkway wearing two different shoes (Figure 1): first wearing model A and then model B, with different constructive characteristics and purposes. Model A (Newfeel Soft 140 by Newfeel, Villeneuve-d’Ascq, France) is meant for walking, and has a solid sole made of 100% ethylene vinyl acetate (10 mm thick in the forefoot and 20 mm thick in the heel, shore A 38) and the exterior is a thin fabric made of 100% recycled polyester. Model B (Asics gel Dedicate 5 by Asics Corporation, Kobe, Japan) is meant for playing tennis, and has a more complex sole (13 mm thick in the forefoot and 26 mm thick in the heel, combining a flexible rubber core—shore A 45—and high abrasion rubber—shore A 70—in the inferior part) that is lightened in the middle and reinforced with a carbon truss and incorporates a forefoot gel cushioning system, and the exterior is a synthetic leather.

Each type of shoe was fitted to each participant. For each shoe, the participant had to walk by stepping with the right foot on a pressure platform (to measure contact pressure between the shoe sole and the ground) located in the middle of the walkway while wearing in-shoe pressure sensors (to measure contact pressure between the foot and the shoe sole) for his/her right foot. The participant was instructed to face forward while walking to avoid platform targeting. Five trials were considered, but the participant walked as many times as required to complete five valid trials. When the participant did not step on the platform with his/her right foot, the trial was discarded. Before data collection started, the participants walked on the walkway several times to become familiar with each walking condition.

### 2.3. Kinematic and Kinetic Data Obtaining

**Figure 2:**
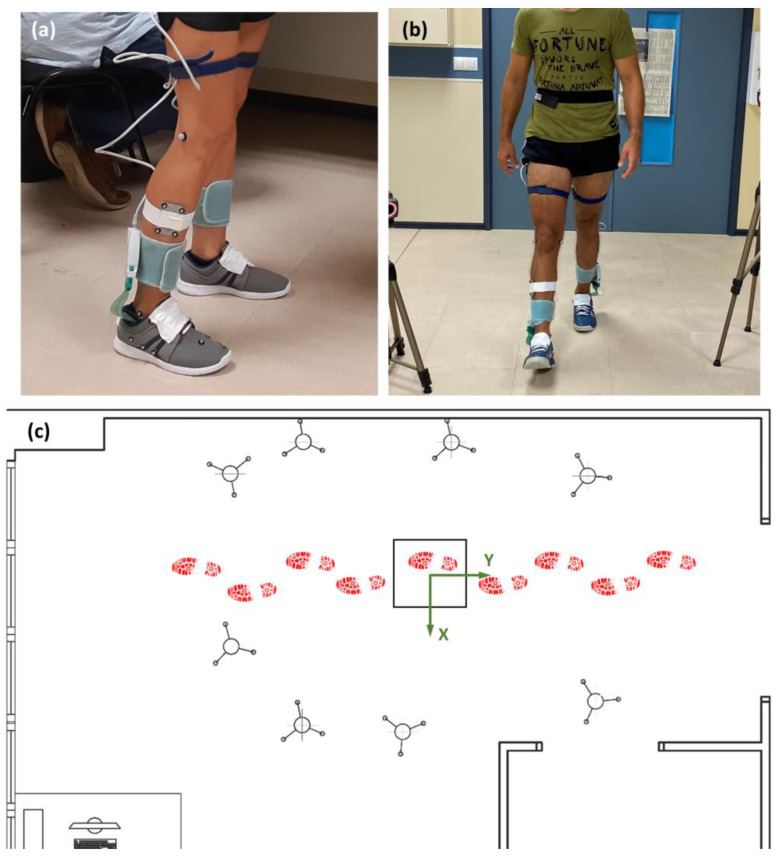
Setup used for the experiment: (**a**) detail of the in-shoe system and markers; markers C1, C2, MC, LC, B1, H2, HX, H1, H3, NV, CU and B5 were applied to the shoe; (**b**) a participant walking during a trial; (**c**) camera and platform arrangement on the walkway and the global coordinate system.

The kinematics of the ankle, MT and MP joints were recorded using an adaptation of the model proposed by Bruening et al. [1], as presented in Sanchis-Sales et al. [36], but with foot markers applied to the shoe from palpation through the shoe upper, as in [37] (Figure 2). Although using markers applied to the shoe involves some error due to foot motion inside the shoe and shoe deformation, it has the advantage of not altering the shoe, avoiding unrealistic behavior, and allowing shoe reuse for other participants (with the same size). The position and orientation of segments (shank, hindfoot, forefoot, and hallux) were tracked at 100 Hz using reflective markers and an 8 infrared camera motion analysis system (Vicon Motion Systems Ltd., Oxford, UK). Joint angles were calculated from the upright standing static reference posture using a Cardan rotation sequence between the distal and proximal segments: 1—dorsiflexion/plantarflexion; 2—abduction/adduction; and 3—inversion/eversion [38]. The upright standing static reference posture was recorded to each participant before the gait trials per walking condition (i.e., each shoe model). All the kinematical data were low-pass-filtered using a fourth-order Butterworth filter with a cut-off frequency of 10 Hz.

Contact pressures were recorded with a 0.40 m × 0.40 m Podoprint pressure platform (Namrol Group, Barcelona, Spain; 1 sensel/cm^2^, 4 kPa–1000 kPa) and the in-shoe F-Scan mobile system (Tekscan Inc., Boston, MA, USA; 3.9 sensels/cm^2^, 4 kPa–862 kPa) [39], which were synchronized with the infrared camera system to measure contact pressure at a 100 Hz sampling rate. Care was taken to cut the insole to fit each participant’s shoe. The pressure of each contact cell was applied to the appropriate foot segment by comparing contact cell coordinates with the anteroposterior location of joint centers (e.g., cells with anteroposterior-coordinate between those of the MT and MP joint centers were assigned to the forefoot segment). The global coordinates of each platform cell were known and fixed during the trial because the Vicon global coordinate system was set with the origin at the center of the pressure platform, with the *Y*-axis pointing in the anterior–posterior direction, the *X*-axis in the lateral-medial direction and the *Z*-axis pointing upwardly from the ground. The global coordinates of the in-shoe cells were computed by matching the coordinates of the CoP of the in-shoe and the platform after 0.1 s since the first initial contact, and considering the foot angle with the anterior–posterior axis calculated from markers C1 and H2.

The normal component of the reaction forces and the CoP were calculated for each frame on every foot segment, both for the pressure platform and the in-shoe system, by considering the pressure at each sensing cell and the cell area for each device. The 3D joint moments at each joint were calculated from them (thus, the effect of the weight of the foot and that of foot angular velocity and linear and angular accelerations was neglected [40,41,42]) as the cross product of the ground reaction forces on distal segments to the joint, and the 3-D distances between the corresponding centers of pressure and the joint centers of rotation (as defined by [6]), and were expressed in relation to the local coordinate system of the proximal segment. The joint flexion moments were presented as a percentage of the stance phase in the gait cycle and amplitudes were normalized to body weight. All the kinetic data were low-pass-filtered using a fourth-order Butterworth filter with a cut-off frequency of 50 Hz. The computation of reaction forces and joint moments and filtering was performed with custom-developed software in Matlab version R2022b (The MathWorks Inc., Natick, MA, USA).

### 2.4. Data Analysis

The dorsiflexion/plantarflexion, abduction/adduction and inversion/eversion joint moments were averaged across trials for each participant per condition (i.e., each shoe and every pressure device). Then, the mean dorsiflexion/plantarflexion, abduction/adduction and inversion/eversion joint moments across subjects were computed and plotted versus time, along with the 95% confidence interval. These plots were used to identify the parameters that describe the moment curves on each plane, i.e., the magnitudes and times for the different peaks on the moment curves. Then, these descriptive parameters were computed for each participant for each of the five trials under every condition (a total of 200 values per parameter—10 subjects × 2 shoes × 2 devices × 5 repetitions). These parameters were used as the dependent variables in a set of mixed-effect models (one per parameter) with fixed and random effects, aiming to study the influence (*p* < 0.05) of the device on the kinematic parameters. Given the structure of the data (patients walked 5 times in 2 shoe conditions, and kinematic parameters computed using data from 2 devices) it was considered necessary to build the model by inserting the fixed factors ‘device’, ‘shoe’ and their interaction, and a random intercept and slope relating to the subjects participating in the experiment, considering also repeated measures for each interaction ‘subject × shoe × device’. Given the moderate sample size, and that the model assumptions were violated (normality), the restricted maximum likelihood was used as estimation method. Scaled identity and Toeplitz covariance structures were assumed for random factor and residues, respectively, based on Akaike’s information criterion. These tests allow for a comprehensive analysis, where the influence of using one or other device can be evaluated through significance (*p* < 0.05) of factor ‘device’, but can be also complemented by examining whether the device affects all shoes in the same way through significance of factor ‘device × shoe’. Finally, test–retest reliability was analyzed by means of intraclass correlation coefficients for each parameter, which were calculated based on a mean-rating (k = 5), 2-way mixed-effects model, and both absolute agreement and consistency. All the statistical analyses were performed using SPSS statistical package version 28 (SPSS Inc., Chicago, IL, USA).

## 3. Results

**Figure 3:**
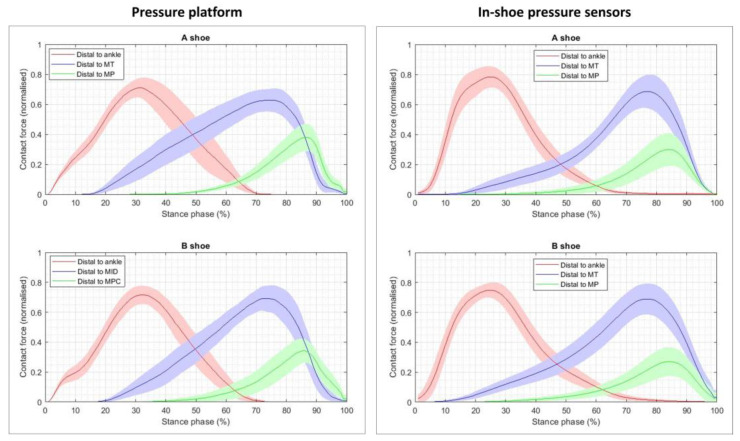
Plots showing the mean and 95% confidence interval of the segmented normal contact forces versus time for each shoe condition (A shoe and B shoe), averaged across all the subjects and trials. Left, the plots obtained from the pressure platform data; right, from the in-shoe pressure sensors’ data.

Figure 3 shows the mean and 95% confidence interval of the segmented normal contact forces (distal to each joint) versus time for each of the shoe conditions (A shoe and B shoe), recorded with the pressure platform data and with the in-shoe pressure sensors’ data. The curves show a bell-shaped profile, which is similar across shoe conditions and recording devices, so that each could be described by its maximal value (MaxVal) and the time instant at which it occurred (TMax).

**Figure 4:**
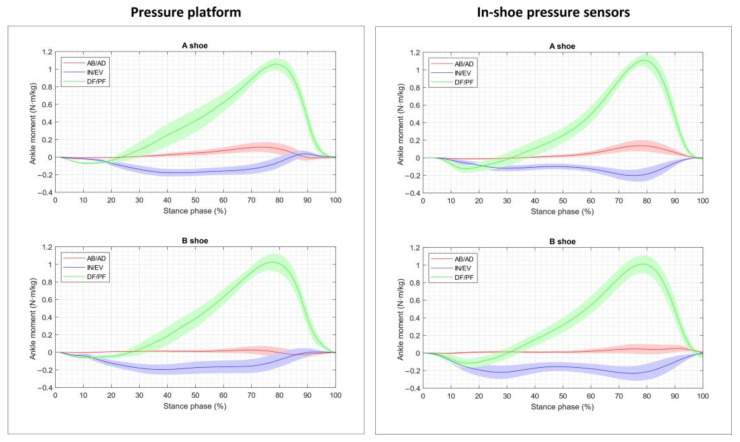
Plots showing the mean and 95% confidence interval of the dorsiflexion/plantarflexion (DF/PF), inversion/eversion (IN/EV) and abduction/adduction (AB/AD) moments at the ankle joint versus time for each shoe condition (A shoe and B shoe), averaged across all the subjects and trials. Left, the plots obtained from the pressure platform data; right, from the in-shoe pressure sensors’ data.

**Figure 5:**
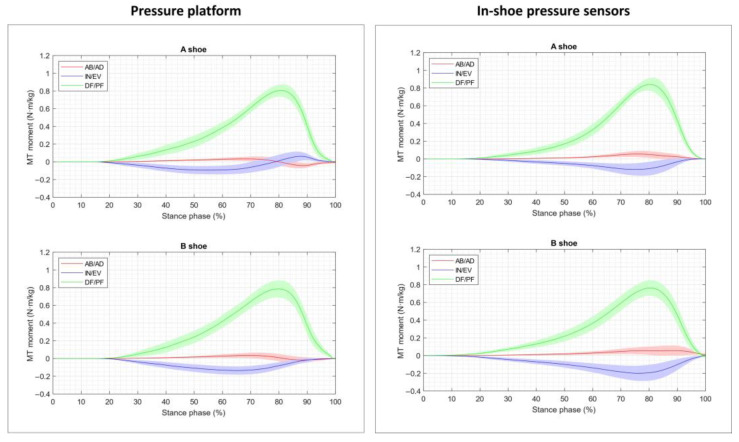
Plots showing the mean and 95% confidence interval of the dorsiflexion/plantarflexion (DF/P), inversion/eversion (IN/EV) and abduction/adduction (AB/AD) moments at the MT joint versus time for each shoe condition (A shoe and B shoe), averaged across all the subjects and trials. Left, the plots obtained from the pressure platform data; right, from the in-shoe pressure sensors’ data.

**Figure 6:**
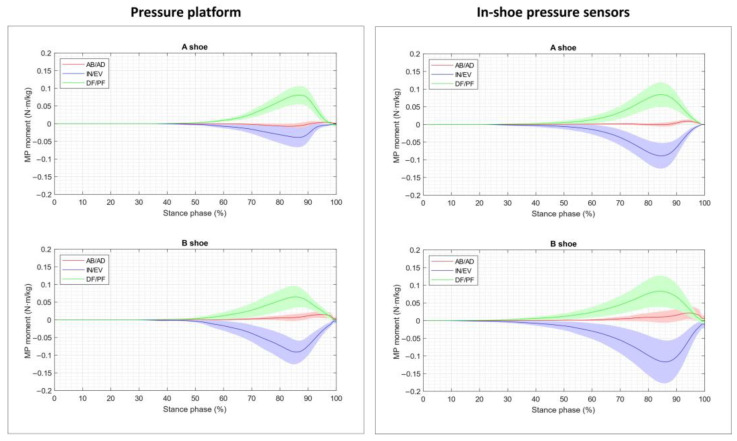
Plots showing the mean and 95% confidence interval of the dorsiflexion/plantarflexion (DF/PF), inversion/eversion (IN/EV) and abduction/adduction (AB/AD) moments at the MP joint versus time for each shoe condition (A shoe and B shoe), averaged across all the subjects and trials. Left, the plots obtained from the pressure platform data; right, from the in-shoe pressure sensors’ data.

Figure 4, Figure 5 and Figure 6 depict the corresponding moment data obtained from the segmented contact forces for each joint and in each motion direction (note that the scale for the MP moment data is larger than that used for the ankle and MT joints). The abduction/adduction moments were very low throughout the stance phase in all the joints, and they were not considered for the comparison in the nonparametric tests. For the dorsiflexion/plantarflexion moment curves, MaxVal and TMax were considered, while the minimal value (MinVal) and the time instant at which it occurred (TMin) were contemplated for the inversion/eversion moment curves.

Table 1 shows the significant levels (*p*-values) obtained in the mixed-effect models on the parameters describing contact forces, for each considered factor. Table 2, Table 3 and Table 4 indicate the significant levels for the parameters describing the corresponding moment curves for each joint in each motion direction.

No significant differences were found for the factor ‘shoe’ in the contact force parameters, and only two of the twelve moment parameters showed significant differences.

Significant differences were detected only in two contact force parameters depending on the used device, although other two parameters were close to significance. However, all the moment parameters of the ankle, MT and MP joints did not show any significant differences, except for the time when the peak was observed in the inversion/eversion moment at MT joint, and the peak time for dorsiflexion/plantarflexion at the MP joint. Furthermore, the effect of the device was consistent, as it affected both shoe conditions in the same way (*p* > 0.05 for factor ‘device × shoe’).

Finally, Table 5 shows the intraclass correlation coefficients for the moment parameters obtained with the two recording devices. Their significance is marked in bold. All the correlation coefficients were significant (*p* < 0.05), except for the time instant corresponding to inversion/eversion peak value occurrence. The correlation coefficients were all below 0.84.

## 4. Discussion

This work provides detailed data that describe the reactive forces and moments at the ankle, MT and MP joints of healthy subjects’ feet during in-shoe walking with two different footwear types, obtained by simultaneously recording pressure data with two different devices: instrumented insoles, which provide information about foot sole contact forces, and pressure platforms, which supply information about contact forces between the shoe sole and the ground.

Broadly speaking, the curves obtained for both in-shoe conditions with the two pressure devices were similar. The ground reaction forces generated the highest moments on the sagittal plane (up to 1.2 N·m/kg DF moments), followed by the frontal plane (up to 0.2 N·m/kg EV moments). The MP joint was significantly less stressed than the ankle and MT joints, and the moments on the transverse plane were very small. These profiles were similar to those reported in previous works for the barefoot condition [6,11,13], although the peak values were somewhat lower: (i) the peak joint moments were approximately 15% lower than those measured with force plates in [6,13], which can probably be attributed to the frictional forces; (ii) when measured with pressure platforms [11], slightly lower peaks were observed for the DF moment at the ankle and MT joints, which were achieved at a slightly later stance time (closer to 80% instead of to 75%, especially at the MT joint), which indicates the cushioning effect of the shoe (when compared to previous authors’ data, obtained using the same model but under barefoot conditions). Comparing other in-shoe studies was only possible for the ankle joint. When carrying this out, this delay in the peak time agreed with the results obtained at the ankle for the in-shoe gait reported in [35], although the reported peak moments were higher (approximately 20%) than those found herein and, once again, can be probably attributed to frictional forces.

The results of the mixed-effect tests enabled us to perform a more in-depth analysis of the factors considered in this study. The shoe condition significantly affected the foot kinetics (the factor ‘shoe’ was significant) regarding inversion/eversion joint moments at the MT and MP joints, with a lower peak for the most flexible and lightweight shoe. These results mean that care must be taken when comparing the in-shoe kinetic data from different studies, seeing that specific shoe features affect the dynamic response (foot joint moments). In this particular case, the characteristics of the model A shoe constrained the foot movements less than the model B shoe, and also the contact was less modified because of the complexity of the sole of the model B shoe, so that the inversion/eversion peak moment values were closer to those reported in previous works for the barefoot condition.

When focusing on device details, significant differences were only obtained for the peak times of ground reaction forces at the ankle and MT joints, although the corresponding peak values were close to significantly different (the mean difference between the mean peaks was 10.8%). The final effect on joint moments was more noticeable at the MP joint (26%) and was lower for the ankle (8.2%) and MT joints (15%), but significant differences were only obtained for the peak times of inversion/eversion at the MT joint and dorsiflexion/plantarflexion at the MP joint. The differences in ankle moments obtained (although nonsignificant) when using instrumented insoles instead of pressure platforms could be attributed to the fact that insoles do not provide all the contact forces that act on the foot. The device seemed to affect both the considered shoe conditions in the same way (factor ‘device × shoe’, nonsignificant). These findings suggest the interchangeability of measuring joint moments with one or other device, independently of the footwear condition. This hypothesis was reinforced by the intraclass correlation coefficients obtained, especially for the peak values, which presented higher values (although only moderate-to-good reliability) than the time when they occurred (poor-to-moderate reliability).

## 5. Study Limitations

Nevertheless, the present study has some limitations. Calculated joint moments did not take into account contact frictional forces because no force plates were used; more research is needed to implement a method to estimate contact friction forces when using instrumented insoles. Joint moments were computed from reaction forces measured with pressor sensors, which are less accurate than force plates. Using markers applied to the shoe involves some (uncertain) error in the kinematics measurement due to foot motion inside the shoe and shoe deformation. Furthermore, the results were limited by only describing healthy adult kinetics during gait and the two considered shoes. The shod condition measurement with the pressure platform was not specific for foot surface but for the shoes outsole, therefore modifying the shoes means to modify the shoes outsole and the relative contact surface, which may lead to a possible modification of the present results. Other activities (running, jumping, etc.) might present different patterns, which should be investigated. Sensitivity of results to in-shoe insole positioning has not been studied. Finally, the presented results are restricted by the employed model, and only a direct comparison is possible with only the data obtained from using an analogous model and reference posture.

## 6. Conclusions

The study provides novel quantitative data on the differences in the foot joint moments (ankle, MT and MP joints) during in-shoe gait arising from considering foot sole contact forces (measured with instrumented insoles) vs. ground contact forces (measured with a pressure platform). The pros and cons of using these two alternatives can be summarized as follows:

1. Both managed alternatives have drawbacks: the platform does not provide direct information about the contact forces that act on the foot, and the instrumented insoles do supply direct information, but it is incomplete (as shown from the differences in ankle moments).
2. Recording ground reaction forces has the advantage of enabling contact friction forces to be considered by either directly using force plates in series, as in [6], or combining data from a pressure platform and a force plate to estimate friction forces and torque, as in [15]. Moreover, this alternative is less invasive than using instrumented insoles, which may affect the subject’s gait.
3. In any case, applying any of the alternatives is plausible if the necessary precautions are taken. Further research should focus on developing methods to translate the kinetic data obtained from both alternatives to make the results taken from different literature studies comparable.

The results of this study highlight the need for further research into the development of sensors that enable the in-shoe kinetic analysis of foot joints under conditions that are as realistic as possible and to place special emphasis on the recording of friction forces and torque.

## Figures and Tables

**Table 1:** Statistical significance levels (*p*-values) obtained for the contact force parameters for all the factors in the mixed-effect models. Statistically significant values (*p* < 0.05) are highlighted in gray and marked in bold.

Joint	Parameter	Device	Shoe	Device × Shoe
Ankle	MaxVal	0.065	0.378	0.550
	TMax	**<0.001**	0.856	0.598
MT	MaxVal	0.699	0.386	0.639
	TMax	0.220	0.988	0.812
MP	MaxVal	0.057	0.843	0.856
	TMax	**0.012**	0.324	0.607

**Table 2:** Statistical significance levels obtained for the ankle moment parameters for each factor in the mixed-effect models. No statistically significant values (*p* < 0.05) were observed. Abbreviations: inversion/eversion (IN/EV), dorsiflexion/plantarflexion (DF/PF).

Motion	Parameter	Device	Shoe	Device × Shoe
Ankle IN/EV	MinVal	0.322	0.123	0.828
	TMin	0.167	0.218	0.725
Ankle DF/PF	MaxVal	0.655	0.302	0.495
	TMax	0.577	0.464	0.735

**Table 3:** Statistical significance levels obtained for the MT moment parameters for each factor in the mixed-effect models. Statistically significant values (*p* < 0.05) are highlighted in gray and marked in bold. Abbreviations: inversion/eversion (IN/EV), dorsiflexion/plantarflexion (DF/PF).

Motion	Parameter	Device	Shoe	Device × Shoe
MT IN/EV	MinVal	0.096	**0.024**	0.607
	TMin	**<0.001**	0.191	0.819
MT DF/PF	MaxVal	0.906	0.496	0.566
	TMax	0.903	0.930	0.561

**Table 4:** Statistical significance levels obtained for the MP moment parameters for each factor in the mixed-effect models. Statistically significant values (*p* < 0.05) are highlighted in gray and marked in bold. Abbreviations: inversion/eversion (IN/EV), dorsiflexion/plantarflexion (DF/PF).

Motion	Parameter	Device	Shoe	Device × Shoe
MP IN/EV	MinVal	0.162	**0.021**	0.496
	TMin	0.393	0.077	0.736
MP DF/PF	MaxVal	0.659	0.856	0.764
	TMax	**0.002**	0.846	0.172

**Table 5:** Consistency (and absolute agreement between parenthesis) intraclass correlation coefficients for the parameters obtained with the in-shoe pressure sensors and the pressure platform. Statistically significant values (*p* < 0.05) are marked in bold.

Joint	Inversion/Eversion		Dorsiflexion/Plantarflexion	
	MinVal	Tmin	MaxVal	Tmax
Ankle	**0.836 (0.828)**	**0.285 (0.275)**	**0.693 (0.693)**	**0.612 (0.610)**
MT	**0.712 (0.688)**	**0.301 (0.250)**	**0.695 (0.697)**	**0.471 (0.473)**
MP	**0.790 (0.771)**	**0.807 (0.800)**	**0.817 (0.815)**	0.261 (0.209)

## Data Availability

The experimental data are publicly available at Zenodo (https://doi.org/10.5281/zenodo.7970419).
